# Oxidative Stress and Its Role in the Emergence and Progression of Myelodysplastic Syndromes: Insights from Proteomic Analysis and Other Methodologies

**DOI:** 10.3390/proteomes13020021

**Published:** 2025-06-03

**Authors:** Anastasia Boura-Theodorou, Konstantina Psatha, Stefania Maniatsi, Areti Kourti, Georgia Kaiafa, Michalis Aivaliotis, Kali Makedou

**Affiliations:** 1 Laboratory of Biochemistry, AHEPA General Hospital, School of Medicine, Faculty of Health Sciences, Aristotle University of Thessaloniki, GR-54124 Thessaloniki, Greece; ampourath@auth.gr (A.B.-T.); aretikourti@auth.gr (A.K.); 2 Functional Proteomics and Systems Biology (FunPATh), Center for Interdisciplinary Research and Innovation (CIRI–AUTH), GR-57001 Thessaloniki, Greece; kpsatha@auth.gr (K.P.); maniatsi@auth.gr (S.M.); aivaliotis@auth.gr (M.A.); 3 Laboratory of Biological Chemistry, School of Medicine, Faculty of Health Sciences, Aristotle University of Thessaloniki, GR-54124 Thessaloniki, Greece; 4 Laboratory of Medical Biology—Genetics, School of Medicine, Faculty of Health Sciences, Aristotle University of Thessaloniki, GR-54124 Thessaloniki, Greece; 5 1st Propedeutic Internal Medicine Department, AHEPA General Hospital, School of Medicine, Faculty of Health Sciences, Aristotle University of Thessaloniki, GR-54124 Thessaloniki, Greece; gdkaiafa@auth.gr; 6 Basic and Translational Research Unit, Special Unit for Biomedical Research and Education, School of Medicine, Aristotle University of Thessaloniki, GR-54124 Thessaloniki, Greece

**Keywords:** myelodysplastic syndromes, oxidative stress, proteomic analysis, bone marrow plasma, serum

## Abstract

Myelodysplastic syndromes (MDS) belong to a category of malignant stem-cell and myeloid disorders that deteriorate the function of the hematopoietic system exacerbated by the omnipresent anemia that characterizes myelodysplasia. The pathogenesis of MDS is driven by cytogenetic abnormalities along with the excessive production of pro-inflammatory cytokines and disruptions in inflammatory signaling pathway, particularly through the influence of carbonylated proteins, which are linked to MDS progression. An additional and major contributor to the pathogenesis of MDS is oxidative stress marked by uncontrolled levels of reactive oxygen species (ROS), which have been suggested as potential biomarkers for assessing disease severity and stratifying MDS cases throughout a variety of methods. Excessive and non-accumulative levels of free iron can also lead to iron overload (IOL)—related promotion of a high oxidative state, whether we refer to treatment-related IOL or natural IOL mechanisms. Proteomic analysis has emerged as a powerful tool for profiling protein samples, and, consequently, understanding the molecular changes underlying MDS. In this review, we evaluated studies and their methodologies aiming in investigating distinctive proteomics signatures associated with MDS pathogenesis, focusing on the role of oxidative stress at the protein level.

## 1. Introduction

The myelodysplastic syndromes (MDS) are a group of heterogeneous hematological neoplastic disorders of the bone marrow, characterized by ineffective hematopoiesis, variable cellularity of the bone marrow and cytopenias [1]. The heterogeneity of the disease is linked to variations in the morphology of peripheral blood and bone marrow, molecular genetic alterations, that account for the majority of the MDS cases, and, in some cases, cytogenetic abnormalities [2,3,4]. Somatic mutations are also carried by MDS, and they can be tracked down with the help of Next Generation Sequencing (NGS). Additionally, cytogenetic analysis has shown that MDS progression is associated with a deficiency in hematopoietic stem cells, a fact observed in approximately 50% of cases [4].

The diagnosis of MDS relies on peripheral blood tests, such as a complete blood count (CBC) and microscopic examination of a peripheral blood smear to detect for morphological abnormalities, as well as a bone marrow biopsy and aspiration, supplemented by with karyotypic and specific molecular testing. The staging of MDS ranges from low-risk subgroups with favorable prognosis and extended survival to high-risk subgroups, which often progress to acute myeloid leukemia and has a poor prognosis. The latter is classified using the International Prognostic Scoring System (IPSS), whereas the World Health Association (WHO) classification defines six different subgroups based on diagnosis. These subgroups serve as a cornerstone for patient assessment and treatment decisions [5].

There are a variety of mechanisms linked to the emergence of MDS. Regarding defective hematopoiesis and the consequent cytopenia, apoptotic pathways contribute significantly to myeloid progenitor dysfunction [6]. Programmed cell death is regulated by both intra- and extracellular reactive oxygen species (ROS), highlighting the pivotal role of oxidative stress in the pathophysiology of this disease. Persistent oxidative stress and the resulting damage to macromolecules (e.g., DNA, proteins) further accelerate disease progression [7].

The investigation of MDS, oxidative stress and their underlying molecular mechanisms is primarily conducted through various analytical methods. Among these, proteomic analysis has emerged as a powerful tool enabling the characterization of the protein profile of a biological sample through integrated methodologies [8]. This review aims to comprehensively examine the existing literature on MDS research, particularly focusing on the significance of proteomic approaches in studying MDS pathogenesis, highlighting its role in studying cellular pathophysiology, molecular mechanisms, and clinical implications. Additionally, we will discuss the various methodologies employed to study oxidative stress in MDS, with a particular emphasis on proteomic techniques.

## 2. Materials and Methods

### Search Strategy and Selection Criteria

A literature search was conducted across PubMed, Scopus, ScienceDirect, ResearchGate, and Frontiers. The keywords used in this study included: myelodysplastic syndromes, oxidative stress, reactive oxygen species, proteomic analysis, oxidatively modified lipoproteins, and biomarkers of oxidative stress in myelodysplastic syndromes in human serum and bone marrow plasma.

The exclusion criteria included studies involving patients receiving any treatment for MDS, pediatric cases, animal studies, in vitro lymphoma cell studies, and genetic studies limited to MDS—related gene identification.

From the initial pool of 150 articles ten met the inclusion criteria and were selected for this review (Table 1).

Our main objective was to explore the protein-based association between MDS and oxidative stress through proteomic analysis. While our focus remained on MDS, we also included select studies on AML, due to the shared pathogenic mechanistic similarities between system deregulation in MDS and its potential progression to AML. Given the limited availability of comprehensive studies combining all relevant factors, these AML-related papers provided valuable supplementary insights justifying their inclusion. Additionally, because of the large number of genetic aberrations observed in MDS, studies examining the interplay between oxidative stress, biomarkers and gene-based methodologies were also incorporated to offer a more holistic perspective.

## 3. Myelodysplastic Syndromes

MDS represent a heterogeneous group of malignant stem cell and myeloid disorders, characterized by ineffective hematopoiesis, cytopenia, and significant dysfunction in the maturation of erythroid, megakaryocytic, and granulocytic lineages. The latter is strongly associated with a high risk of progression to AML [1].

To date, MDS have been observed in both pediatric and adult patients, as established in several reviews [20,21]. However, childhood MDS accounts for fewer than 5% cases diagnosed, making it predominantly a disease of the elderly. De novo MDS—cases that are not associated with prior treatment (therapy-related MDS, t—MDS) or exposure to chemotherapy or radiotherapy—is particularly common in older populations, with 77% of cases occurring in individuals aged 60 and above [22].

WHO introduced the terms CHIP (Clonal Hematopoiesis of Indeterminate Potential) and CCUS (Clonal Cytopenia of Undetermined Significance) to describe pre-MDS myeloid neoplasms that may precede full-blown MDS [23]. Since CCUS and MDS share similarities—such as the advanced age of affected patients, high rates of cytopenias, and recurrent mutations—they are often clinically indistinguishable. For this reason, both the International Consensus Classification (ICC) and the World Health Organization (WHO) classification of lympho–hemopoietic neoplasms (WHO–HAEM5), the fifth edition released in 2022, merged these entities into the same diagnostic category [23,24,25,26,27]. Regarding the new nomenclature [27], MDS is now referred to as myelodysplastic neoplasms (MDN) in order to state clearly the appearance of a neoplastic disorder. Some previous subcategories were consolidated due to overlapping features, while others (e.g., *MDS unclassified*) were eliminated. Table 2 and Table 3 summarize the evolution of the WHO classification systems over the time (2001 [28], 2008 [29], 2016 [27], 2022 [27]).

Although 10–15% of the general population may carry mutations associated with CHIP and CCUS without ever developing MDS, these genetic alterations can play a crucial role in disease progression and influence patient outcomes [30].

### 3.1. Classification of the Myelodysplastic Syndromes

The IPSS [31] originally divides MDS patients into two categories, low and high risk, to classify them according to their clinical course and survival rate. These categories range from a decreased rate of cytopenia and progression to AML, associated with prolonged survival (low-risk MDS) to increased cytopenia rates and the appearance of AML, leading to a significantly worsened prognosis (high-risk MDS) [32].

As previously mentioned, most MDS cases present with cytogenetic and molecular cytogenetic alterations. Therefore, the IPSS was eventually replaced by two more refined systems: IPSS—R (Revised IPSS) and IPSS—M (Molecular IPSS), which provide a much more detailed view of genetic aberrations and cytopenia status [33,34]. The main difference between IPSS and IPSS—R is that the IPSS—R stratifies patients into five subgroups for risk assessment, whereas the original IPSS used only two general categories. Specifically, under the original IPSS, lower-risk disease includes low- and intermediate-1-risk patients, while IPSS—R classifies them as low or some intermediate subsets. On the other hand, IPSS high-risk disease includes intermediate–2 and high-risk patients, whereas IPSS—R spans intermediate, high, and very high-risk groups. Importantly, debate remains regarding which IPSS-R intermediate-risk patients should be considered lower vs. higher risk [35].

Risk stratification was enabled, as two new systems were introduced, the Clonal hematopoiesis risk score (CHR) and the Clonal Cytopenia Risk Score (CCRS) [26,35,36]. The CHRS is an efficient prognostic score model predicting the progression of CHIP/CCUS to Myeloid Neoplasia (MN). This model counts on the presence of CCUS, mutation patterns, patient age, red blood cell indices, and other factors. Individuals with a CHRS of ≥12.5 are considered high risk (high chance of progressing to MN). In contrast, those with a CHRS between 10 and 12 are considered intermediate risk, and individuals with CHRS of <9.5 are considered low risk (low chance of progressing to MN). The research group of Xie Z., et al. [26], proposed the CCRS model. According to their work they have established a three-category patient group, where CCRS scores < 2.5 are linked with low-risk patients, CCRS 2.5 to <5 with intermediate risk patients and CCRS ≥ 5 with high-risk patients. Matos A, et al. [30] reviewed and demonstrated that in cases of low severity MDS, cell death and elevated cytokine levels, which are major drivers of tumor development, were correlated with an increased initiation rate of the disease. In contrast, higher disease severity was associated with lower rates of programmed cell death.

The 2016 WHO classification introduced several MDS subtypes, including: MDS with single lineage dysplasia (MDS–SLD), MDS with multilineage dysplasia (MDS–MLD), MDS with ring sideroblasts (MDS–RS), MDS with excess blasts I and II (MDS–EB I, II), MDS with deletions in the chromosomes 5 [MDS–del(5q)] and 7 [MDS–del(7q)], and MDS unclassified (MDS–U) [37]. For the MDS–RS subtype, in particular, it is essential to distinguish between single-lineage and multilineage dysplasia, resulting in the terms MDS–RS with single lineage dysplasia and MDS-RS with multilineage dysplasia [38]. In 2022 the WHO–HAEM5 was introduced as mentioned above. For a thorough examination we created Table 3, in which we present the different classification systems throughout the years.

Anemia plays a key role in the majority of MDS subtypes, affecting up to 80% of cases. The type of anemia observed in MDS involves insufficient erythropoiesis, mild hemolysis, a low reticulocyte count, and persistence despite treatment, leading to the term “refractory anemia”. As a result, some subtypes have been renamed. These include refractory anemia with excess blasts I and II (RAEB–I, RAEB–II), with 5–9% and 10–19% blasts, respectively [39]. The term “refractory cytopenia” is used for cases where cytopenia is dominant, such as refractory cytopenia with multilineage dysplasia (RCMD).

As discussed, the terminology for these subtypes refers only to the term MDS and the specific subtype. This is because, according to the 2016 WHO classification, some studies have shown that anemia is not always persistent despite treatment. Moreover, the WHO classification primarily relies on the morphological features of cells and the number of blasts. In most cases, lower levels of red and white blood cells and platelets do not significantly alter the MDS classification [38]. Nevertheless, not all research groups avoid using the term “refractory anemia” in their studies.

### 3.2. MDS Pathogenesis

The heterogeneity of MDS is attributed to morphological differences in the peripheral blood (PB) and marrow (BM), as well as to cytogenetic abnormalities, which are found in 50% of cases through karyotypic analysis [40]. Routine analysis of PB contributes to the diagnosis of MDS, alongside BM biopsy, as these tests provide a clear image of morphology, blast percentage, and the number and type of cells. Dysplasia, in combination with cytopenia, contributes to diagnosis [41].

Defects in both the innate and adaptive immune systems play a significant role in the pathogenesis of MDS. Dysregulation of Toll-like receptors (TLRs), which are responsible for the appearance of pro-inflammatory cytokines, leads to excessive activation, driving the system into pyroptosis, a form of inflammatory programmed cell death [42]. Pyroptosis, along with apoptosis, is a key factor in the development of cytopenia, as both processes involve the activation of initiator caspases, which initiate a cascade of caspases that lead to cell death. In pyroptosis, the activated caspases–1, 4, and, 5 in humans, and caspase–11 in mice produce IL–1β and IL–18 which signal the onset of pyroptosis. Conversely, apoptotic bodies are formed when caspases–8 and –9 are activated, which in turn activate pro-caspases–3/7, leading to the activation of caspases–3/7 [43,44].

Cytokines are generally susceptible to chronic inflammation, which accelerates the progression of MDS. Whether in the blood or bone marrow, the number of cytokines steadily increases and significantly contributes to MDS progression, as they play a role in initiating the apoptotic process. The prevalence of programmed cell death correlates with the severity of MDS and directly impacts the overall survival of the patient, as previously discussed [30].

Gene aberrations are another crucial factor in MDS progression and include the expression of signal transporters, which further dysregulate TLR dynamics [30,45,46]. Genetic mutations in MDS are characterized by genetic material methylation, chromatin modification, RNA splicing, DNA repair, and signal monitoring. Interestingly, disease-free individuals often exhibit similar characteristics. An explanation to this was given after Jaiswal S [47] et al. and Genovese G et al. [48] proved that this case is attributed to age and has a high prospect of evolving to MDS or other BM disorders, as it signifies, in its majority, a precancerous state.

Cytogenetic alterations are significant in MDS, also because they are strongly linked with the loss or acquisition of a great number of chromosomes, leading to cases of del (5q), del (7q), monosomy 7, i (17q), trisomy 8 and del (20q), contributing significantly in MDS pathogenesis [49,50]. Del (5q), del (7q) and i (17q) are the aberrations mostly obtained in MDS patients and have a diagnostic advantage regarding some other abnormality-linked MDS cases because they may confirm the disease without the existence of dysplastic features [51].

## 4. Oxidative Stress

Oxidative stress is a state of imbalance between ROS, leading to a state of high rate of DNA rupture and genetic abnormalities. ROS are a group of highly reactive molecules, including oxygen molecules (O_2_), superoxide anion radicals (O^•−^), hydroxyl free radicals (•OH), singlet oxygen (^1^O_2_), and hydrogen peroxide (H_2_O_2_). There are various sources of ROS, both intracellular and extracellular. Intracellular ROS production is primarily driven by mitochondrial and enzymatic processes, with mitochondria contributing significantly (1–5%) as the main source of ROS. External factors, such as oxygen exposure (O_2_) and ultraviolet (UV) radiation, also contribute to the emergence of oxidative stress [52].

**Figure 1:**
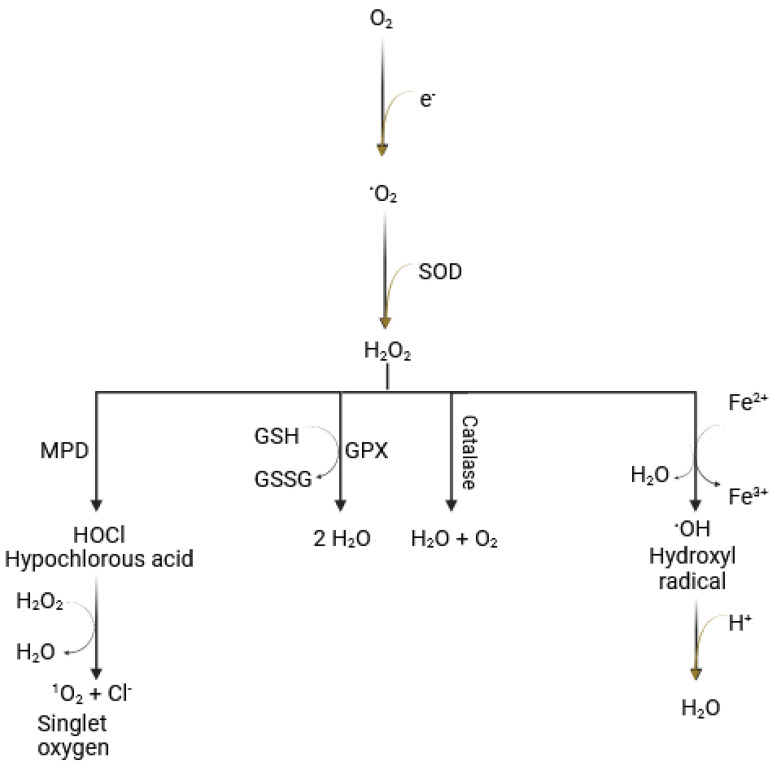
Reactive oxygen species and Fenton reaction. The reduction of molecular oxygen (O_2_) to water is essential for cellular activity and the production of aerobic energy. Simultaneously, the resulting superoxide (O^•−^) is converted to hydrogen peroxide (H_2_O_2_) by the enzyme superoxide dismutase (SOD). The Fenton reaction involves the conversion of hydrogen peroxide to highly reactive hydroxyl free radicals (•OH), mediated by Fe^2+^ (Figure originally created by the authors).

Under normal physiological conditions, ROS are produced when molecular oxygen partially incorporates electrons, leading to the generation of superoxide, a process that occurs primarily in the mitochondria. Enzymatic pathways can also generate ROS. For instance, superoxide dismutase (SOD) converts superoxide to hydrogen peroxide (H_2_O_2_), and myeloperoxidase (MPD) produces hypochlorous acid (HOCl). A critical process in ROS generation is the Fenton reaction. In the presence of Fe^2+^, hydrogen peroxide is converted into the highly reactive hydroxyl radical [53], which is the most reactive of all oxygen radicals (Figure 1). The latter plays a major role in oxidizing proteins, lipids (leading to lipid peroxidation), carbohydrates (resulting in advanced glycation end-products (AGEs), and nucleic acids, ultimately causing mutations in DNA and RNA [7].

As mentioned earlier, ROS play a vital role in cellular processes, contributing significantly to cell homeostasis. However, to prevent pathological conditions that could ultimately lead to the patient’s death, ROS must be effectively neutralized by antioxidants [54]. Maintaining balanced ROS levels within the cellular system is crucial for the proper functioning of cell proliferation, differentiation, and self-repair. The development of certain hematological diseases, particularly those involving significant bone marrow dysfunction, has been linked to excessive ROS production and the reduced resilience of hematopoietic cells. A simple explanation for this is that more easily affect hematopoietic cells with lower resistance. The complexity arises when ROS target essential cellular components, including proteins, lipids, and non-coding RNAs, leading to cellular degeneration [16].

### 4.1. Protein Carbonylation and System Deregulation

The combination of protein denaturation, permanent carbonylation (which adversely affects signaling pathways), and the loss of key protein functions contributes to the progression of MDS [16]. Oxidatively modified proteins, which result from the direct attachment of carbonyl groups to the side chains of amino acids such as arginine, proline, lysine and threonine—a process facilitated by metal ions—can confirm this phenomenon. Additionally, a non-direct pathway also contributes to protein carbonylation, where nucleophilic amino acid side chains react with byproducts of lipid peroxidation. Lipid peroxidation is exclusively driven by ROS, and examples of its byproducts include the highly toxic 4-hydroxynonenal (HNE) and the mutagenic malondialdehyde (MDA). Understanding the degree of protein oxidation and thus carbonylation, is crucial because it reflects the extent of oxidation damage in various malignancies and provides valuable insights into the patient’s prognosis.

### 4.2. The Case of Iron Overload (IOL)

With aging, combined with inefficient erythroid cell production and the rupture of cells, there is an abnormal, excessive accumulation of iron that induces a highly oxidative state. This is due to irregular erythropoiesis, in which growth differentiation factors, such as growth differentiation factor 15 (GDF15)—a member of the TGFβ family—shows higher activity, leading to increased intestinal iron absorption in patients diagnosed with RARS [55]. The resulting environment can be detrimental to various organs. In the liver, the production of hepcidin, which is iron-regulated and responsible for iron accumulation in plasma, is suppressed. The function of the hematopoietic system, particularly in patients with low/intermediate–1 risk myelodysplasia, is also significantly impaired by oxidative stress induced IOL [56].

Systemic IOL is not the only cause of oxidative stress resulting from excessive iron accumulation. A specific case arises in transfusion—dependent IOL, which occurs after anemia treatment in the early stages of MDS. IOL is strongly associated with a high likelihood of MDS progression to AML and poor survival rates. When IOL is established [56], transferrin, which normally binds and stores iron, loses its binding capacity. As a result, non-transferrin-bound plasma iron (NTBI) and its reactive redox forms, such as labile plasma iron (LPI), promote ROS production and exhaust antioxidant molecules, further contributing to the pathogenesis of MDS.

### 4.3. Molecular Basis of Oxidative Stress Involvement in MDS

Cells can protect themselves from the increase in ROS that leads the system to a pathological state. To achieve maximum protection, cells assemble a combination of enzymatic and non-enzymatic antioxidants. Enzymatic antioxidants include SOD, MPD, and glutathione peroxidase (GPX), while non-enzymatic antioxidants consist of reduced glutathione (GSH), α-tocopherol, and vitamin C [57,58,59]. In a neoplastic state, the cell’s defense mechanisms must cope with an excessive presence of ROS, which promotes an oxidative stress environment [10]. Cancer progression is driven by ROS at both genetic and epigenetic level [32]. Abnormal DNA methylation, an epigenetic mechanism—whether hypermethylation or hypomethylation—along with mutations in epigenetic regulator genes (e.g., EZH2 and DNMT3A), have been observed in myeloid malignancies, particularly in MDS.

DNA hypermethylation is notably present in cases in which MDS progresses to AML. Gonçalves, A. C et al. [10], and Gonçalves, A. C. et al. [11], investigated the combination of oxidative stress and high rates of DNA and amino acid methylation. ROS exert immunosuppressive action, and they are produced by NADPH oxidase, NOX2, expressed in phagocytic cells [60,61]. An uncontrolled production of ROS by NOX2 and NADPH subunits [of the membrane -flavocytochrome b_558_, (which includes gp91^phox^ and p22^phox^) and cytosolic (p47^phox^, p40^phox^, p67^phox^ forming a complex altogether)] is linked to oxidative stress. The study concluded that oxidative stress plays a significant role in low-risk MDS patients, influencing tumor suppressor gene methylation and contributing to the development of myeloid malignancies. Additionally, GonçalvesA. C et al. [62] and Tsamesidis I. et al. [12] apart from demonstrating the relevance of MDA, rates of peroxides, superoxides, glutathione and ROS with the emergence of the MDS, they proposed that proteomics signatures of oxidative stress can be a prognostic tool. In total these studies managed through their experiments to prove that oxidative stress is not only related to MDS in general, but in particular to different MDS subtypes.

## 5. Proteomic Analysis

Proteomic analysis has emerged as a powerful suite of analytical techniques for comprehensive protein characterization in different biological systems [8,63]. The proteome represents the total number of proteins that are expressed in a biological sample under specific conditions and proteomics are responsible for their identification and characterization, their localization, and their interactions. The dynamic nature of the proteome reflects cellular responses to the (micro)environmental transitions of cells and organisms and the underlying pathophysiological conditions. Proteomics encompass protein identification and quantitation, subcellular localization, interaction network analysis and post-translational modification (PTM) profiling [64,65]. Proteomic complexity arises from multiple molecular processes, alternative RNA splicing, and genetic variations [e.g., mutations, Single Nucleotide Polymorphisms (SNPs)]. These processes generate proteoforms, structurally distinct alterations of genetically encoded proteins exhibiting altered tertiary structures, modified functional domains and, binding affinities, and/or differential regulatory properties.

For the identification of proteins and proteoforms, proteomics relies on high resolution, high sensitivity and high accuracy mass spectrometry (MS) platforms. A typical bottom-up proteomics analysis pipeline includes the following three principal steps:

1. Protein preparation and separation: The initial separation of a sample, is achieved via gel or liquid chromatography (LC) techniques. Gel-based methodologies include one-and two-dimensional SDS PAGE, as well as 2D differential gel electrophoresis (2D–DIGE) [66] for the separation of complex protein mixtures, followed by the in-gel or in-solution digestion for MS analysis [67]. Alternatively, LC, including high-performance liquid chromatography (HPLC) and reversed phase high performance liquid chromatography (RP–HPLC) enables high-resolution separation before the introduction of peptides in the MS [68]. Nano-liquid chromatography (n–LC) offers superior sensitivity and efficiency in chromatography, using minimal sample volumes and reducing solvent waste, making it ideal for coupling with MS [69].

2. Ionization: Peptides are ionized into gas–phase ions for MS analysis. Common ionization techniques include: Matrix-assisted Laser Desorption Ionization (MALDI), Surface Enhanced Laser Desorption/Ionization (SELDI) and Electrospray Ionization (ESI). ESI–MS can be used synergistically with several methods, thus enhancing the precision and rate of the identification [67]. This is followed by the introduction of ions into the MS in which they are accelerated according to their mass and charge with the help of an electric or magnetic field of a mass analyzer [70]. The introduction of tandem mass spectrometry (known as MS/MS or MS^2^) has evolved the way we can sequence the peptide and protein amino acid sequence.

3. Bioinformatics: After data acquisition, bioinformatics tools analyze vast amounts of information, comparing the data to online databases to identify peptides and proteins. Bioinformatics also bridges proteomics with “–omics” technologies [71].

### 5.1. Proteomics and the Myelodysplastic Syndromes

Many research groups are studying MDS and how this disease appears, evolves and the way it can be treated. Some of them focus on protein concentration alterations, some on PTMs and others on protein isoform level. On top of that, there exists a variability in sample type, as many researchers focus on animal samples and many others on human samples. In this review studies included involve only human samples. The research group of Májek P et al. [9,14,15] aimed to detect protein modifications in blood plasma and in different subtypes of patients with MDS, covering in this way a vast range of information. Their research led to three publications within three years. Proteomic analysis was used in all three cases. In their first study they suggested new markers found for plasma samples of patients with refractory anemia with excess blasts type I (RAEB–I) while comparing it with those having refractory cytopenia with multilineage dysplasia. Two proteins, leucine–rich alpha–2–glycoprotein (LRAG) and retinol-binding protein 4 were the two significantly altered proteins in the plasma of the patients. The first one is utterly linked to the evolution of the syndrome, and the diminished levels of both of them in combination with defective apoptotic signals, led also to the conclusion that in the case of RAEB–1 there is severe deterioration of the patient’s outcome.

In their second study [14], they examined 26 patient-control plasma samples in MDS patients with refractory anemia (RA) and refractory anemia with ring sideroblasts (RARS). Using 2D electrophoresis and proteomic analysis, and the results of their previous experiments, they found in common alterations in fragments or entirely in the quality of inter-alpha-trypsin inhibitor heavy chain H4 (ITIH4) protein and retinol-binding protein 4. Comparing the PTMs between the two subtypes (RA and RARS) with the help of mass spectrometry based relative label-free quantification, the researchers acknowledge the difference in the resulting modifications of retinol-binding protein 4 in the one subtype and the other. Taking advantage of the very same method, they pointed at the PTMs of alpha–2–HS–glycoprotein, as this protein has a direct connection to the bone marrow growth and was considered as a prominent biomarker for RA and RARS.

In their third study, the particular research group studied in 2014 [15] the most serious subtype of the MDS, as it has great likelihood in the progression to acute myeloid leukemia (AML), the refractory anemia with excess blasts type II (RAEB–II). Blood plasma from 8 patients and 12 control groups were analyzed with isoelectric focusing, SDS PAGE, and MS/MS (HCT ultra–ion–trap mass spectrometer with nano electrospray ionization) coupled to a nano-LC system. They succeeded in proposing new signatures for the MDS. In total, ITIH4 presented similar structural differences in all the subgroups tested in all three studies. A new element was the low plasma abundance of Alpha–2–HS glycoprotein (A2HSG), a protein also presenting major accordance with the bone marrow growth when compared to the non-patient samples. Another protein, the LRAG, was found in high abundance in RAEB–II, it was also observed in the first study and in patients with RAEB–I. LRAG and its altered forms was proposed as a new biomarker of high-risk groups with MDS as it was profoundly increased in plasma samples and it is known for participating in angiogenesis and granulocytic and neutrophil differentiation.

### 5.2. Proteomics and Oxidative Stress in MDS

In the published research of Hlaváčková A et al. [13], the blood plasma of patients versus control was studied for the measurement of the rate of carbonylated proteins that as potential biomarkers could lead to the confirmation of the role of oxidative stress in the pathophysiology of MDS, but also to the syndrome evolution. Samples of patients with RAEB–1,2, RCMD and RARS and negative controls, were analyzed using proteomics analysis after quantification, separation and tryptic digestion of the protein carbonyls. The proteomic analysis included tandem mass spectrometry (HCT ultra–ion–trap mass spectrometer with nano electrospray ionization) coupled to a nano–LC system. After statistical analysis they concluded that patients with RARS had many more carbonylated proteins than non-patients and patients with RCMD, although RCMD refers to a high-risk group. This observation points out the increased oxidative stress in RARS patients and ROS involvement, although the identification of the proteins participating in this state was considered necessary. A sum of 27 distinctive carbonylated proteins were identified, the majority of which participate in the oxidation stress and inflammatory mechanisms, and their dysfunction plays a role in MDS.

Rodriguez-Garcia A et al. [16] on the other hand, investigated the levels of carbonylated proteins in BM and CD34+ cells before and after treatment with an iron chelator called deferasirox (DFX) of patients with RARS, RCMD, RAEB-II, RAEB-I, RCMD-RS, and CMML. CD34+ cells and oxidized erythroblasts are linked with DNA rupture that is also the target of DFX in the article reviewed. Patients with MDS due to their anemic state need frequent blood transfusions, which unfortunately lead to iron-overload conditional on transfusion and thus to excessive production of ROS. In the current study, the researchers tried to evaluate the suppression of carbonylation in combination with the entrapment of Fe–III by DFX, and thus the inhibition of Fe–I I generation, which is the catalyst in the Fenton reaction and consequently results in the production of the hydroxyl radical. Regulation of signal transduction control via the p53/p21 using DFX therapy and thus the minimization of oxidative stress was conducted. At first, they measured the total protein number through the Bradford method. Moving forward, they used 10% SDS–PAGE for 1D & 2D Oxy–Blot, and they continued with one more SDS-PAGE to aspire the full specter of proteins for the imminent mass spectrometry analysis (MS). After tryptic digestion, SwissProt was utilized for the alignment of the remaining peptides to proteins according to their mass, with the help of certain antibodies so that they could be assured of the findings of carbonylated proteins. Additionally, they did a quantitative PCR (qPCR) for the monitoring of p21 mRNA expression. Statistical analysis showed significantly higher levels of carbonylated proteins in patients with MDS and lower in those treated with DFX. Also, they observed highly carbonylated proteins in MDS erythroblasts. After digestion and MS, they also introduced four strongly carbonylated proteins which were: cytoplasmic actin 1, zinc finger protein 846, 14–3–3 protein zeta/delta, L–lactate dehydrogenase (LDH) and A chain. These proteins are eligible as markers for the pathogenesis of MDS, due to their involvement in the arousal of defects in the cell corpus, their abnormal shape and the disease’s evolution. Finally, they proved the link of oxidative stress and the emergence of MDS by targeting the elevated mRNA expression of p21, which in the presence of DFX was minimized and so was the signal transduction initiated by oxidative stress which regulates the cell cycle through the p53 pathway.

Pimková K et al. [17] used blood serum and plasma samples of patients with a large spectrum of types of RA and MDS. This team of scientists used high performance liquid chromatography (HPLC) and capillary electrophoresis with UV detection to estimate total, oxidized and reduced forms of glutathione (GSH) and aminothiols like, cysteine (Cys), homocysteine (HCys) and cysteinylglycine (CG), whose antioxidant role is considered critical in the defense of the patients. They found that in patients with MDS plasma levels of Cys, Hcys, and CG were augmented, plasma levels of total and oxidized glutathione (GSH) and nitrite were highly absent and lastly all forms of CG were elevated in MDS. Although their findings didn’t serve as planned, malonyldialdehyde (MDA), a well-known marker of oxidative stress, was increased. In other words, according to their results, not only did they find antioxidants that are linked with the excessive amount of IOL but, they proved that oxidative stress is a part of the MDS disorder, and not just the aftermath of IOL.

Chai X et al. [18], on the other hand, were more focused on IOL and the generation of ROS, and so they used flow cytometry, Colony-forming cell (CFC) assay, Competitive Repopulation Assay (CRA), Single-cell colony assay, qRT–PCR, and Western blot to estimate the extent to which IOL impairs the bone marrow. They used BM mononuclear cells (BMMNCs), mesenchymal stem cells and peripheral blood plasma as samples. Their results showed that IOL leads to extensive BM impairment, and defective hematopoiesis. Unlike the previous article they concluded, through their experiments on signal transduction pathways, that oxidative stress can be firmly linked to IOL.

Lastly, the observational cross-linked study of de Souza G.F. et al. [19] adds one more source of evidence in the above, as it demonstrates that overproduction of MDA in patients with myelodysplasia, along with IOL, carries high chances of lipid peroxidation. In addition, they concluded that the plethora of iron observed in the study group reinforces the interference of oxidative stress in cell rupture, and consequently the evolution of the MDS.

In the present study, we encountered several limitations. Specifically, the keywords used revealed a lack of studies focusing on the application of proteomic analysis in MDS. In particular, there was a notable scarcity of data addressing oxidative stress in MDS, which was one of the main objectives of this study. Moreover, in an effort to present possible oxidative stress biomarkers in MDS detectable through proteomics, we found that only a limited number of research articles are currently available on this topic. Furthermore, many studies included patients who were already diagnosed and/or receiving treatment or blood transfusion for anemia, making them unsuitable for inclusion. Last but not least, studies applying proteomic approaches to MDS were far fewer compared to those employing genetic and cytogenetic methodologies.

## 6. Conclusions

Myelodysplastic syndromes consist of a large category of malignancies and are heterogeneous. The latter leads to different ways of system deregulation, which promotes several protein or molecular signatures. These signatures can be of assistance in the estimation of the prognosis of MDS. This study reviewed the existing data on possible biomarkers for MDS, in reference to oxidative stress that is acutely related to MDS and its evolution to acute myeloid leukemia. Regarding the latter, carbonylated proteins could be used as possible markers of MDS as they are oxidatively modified and reflect oxidative stress. Other protein biomarkers, such as LRAG and retinol-binding protein 4, have been linked to the evolution of the syndrome, whereas alpha–2–HS–glycoprotein, were connected to bone marrow growth. Iron and particularly iron overload (IOL) are highly concerning signatures as they are established due to both aging features of patients and treatment of anemia. Lastly, the study of elevated ROS levels along with genetic and epigenetic alterations, has led to the emergence of antioxidant enzymes in their reduced or oxidized form as potential markers for the monitoring of the MDS. Nevertheless, the results of those studies can only offer indications for the use of those biomarkers in clinical practice. Further studies are needed to confirm those results and establish the clinical significance of those biomarkers. There seems to be an entire sector of gene and therapy monitoring regarding MDS but when it comes to protein level, although there are many methods available, proteomic analysis and association of oxidative stress with the MDS hasn’t quite made a proper appearance.

As far as we know, there are currently no studies on the combined investigation of PB serum and BM plasma oxidative stress proteomic profile. Such studies could possibly bring out a novel panel of biomarkers useful in clinical practice for early diagnosis of MDS and risk assessment of the evolvement of MDS to AML. Throughout our study, although we found possible biomarkers as aforementioned, their clinical significance has yet to be investigated. This is attributed to the minuscule number of samples as well as the lack of consistency in finding those biomarkers in more than two or three study groups. Therefore, there is a great need for studies that will investigate the proteomic profile in MDS and will clarify possible oxidative stress mechanisms involved.

## Figures and Tables

**Table 1:** Overview of research articles discussed in the review (MDS: Myelodysplastic syndromes; MDA: malonyldialdehyde; AML: Acute Myeloid Leukemia).

Authors	Year	Sample Type	Possible Biomarkers	Association with MDS
Májek P et al. [9]	2012	Blood plasma	Leucine–rich–alpha–2–glycoprotein (LRAG) Retinol-binding protein 4	Progression to AML Poor prognosis
Gonçalves, A. C et al. [10,11]	2021, 2016	Bone marrow peripheral blood (serum, plasma)	Superoxide anion Gene methylation promoters (p15, p16)	Progression to AML Involvement of oxidative stress in MDS
Tsamesidis I. et al. [12]	2019	Blood serum	Vitamin E, malonyldialdehyde	Involvement of oxidative stress in MDS
Hlaváčková A et al. [13]	2017	Blood plasma	Serum albumin Fibrinogen alpha chain Fibrinogen beta chain Fibrinogen gamma chain Serotransferrin Ig alpha–1 chain C region Alpha–2—macroglobulin Ig mu chain C region Haptoglobin Apolipoprotein A-IV Apolipoprotein B-100 Apolipoprotein Complement C1r subcomponent Complement C3 Complement C4—A; Complement C4—B Complement component C9 Hemopexin Ceruloplasmin Inter-alpha-trypsin inhibitor heavy chain Vitronectin Gelsolin C4b-binding protein alpha chain Plasminogen Histidine-rich glycoprotein Clusterin Kininogen–1 Serum amyloid A–1 protein	Pathogenesis of MDS and oxidative stress
Májek P et al. [14]	2013	Blood plasma	Inter–alpha–trypsin inhibitor heavy chain H4 (ITIH4) protein Retinol–binding protein 4 Alpha-2-HS glycoprotein (A2HSG)	Bone marrow morphology Poor prognosis
Májek P et al. [15]	2014	Blood plasma	LRAG, ITIH4, A2HSG	Progression to AML Poor prognosis
Rodriguez-Garcia A et al. [16]	2019	Bone marrow CD34+ cells	Actin cytoplasmic 1 Zinc finger protein 846 14–3–3 protein zeta/delta L-lactate dehydrogenase, A chain p21	Progression to AML Involvement of oxidative stress Pathogenesis of MDS
Pimková K et al. [17]	2014	Blood serum blood plasma	Cysteine (Cys) Homocysteine (HCys) Cysteinylglycine (CG) MDA	Involvement of oxidative stress Pathogenesis of MDS
Chai X et al. [18]	2015	BM mononuclear cells (BMMNCs) mesenchymal stem cells (MSCs) blood plasma	Iron overload	Bone marrow injury
DeSouza G.F. et al. [19]	2014	Peripheral blood	MDA Lipid peroxides	Involvement of oxidative stress Pathogenesis of MDS

**Table 2:** Previous WHO and ICC classification systems.

WHO—2001 [28]	WHO—2008 [29]	WHO—HAEM4 (2016) [27]
-	Refractory cytopenia with unilineage dysplasia (RA, refractory neutropenia, refractory thrombocytopenia	-
Refractory anemia with ringed sideroblasts (RARS)	Refractory anemia with ringed sideroblasts (RARS)	MDS with ring sideroblasts
Refractory anemia with multilineage dysplasia and ringed sideroblasts	-	-
MDS associated with isolated del(5q)	MDS with isolated del(5q)	MDS with isolated del(5q)
-	-	MDS with single lineage dysplasia
Refractory cytopenia with multilineage dysplasia (RCMD)	Refractory cytopenia with multilineage dysplasia (RCMD)	MDS with multilineage dysplasia
Refractory anemia with excess blasts-1 (RAEB–1)	Refractory anemia with excess blasts	MDS–EB–1 (5–9% blasts)
Refractory anemia with excess blasts-2 (RAEB–2)	Refractory anemia with excess blasts	MDS–EB–2 (10–19% blasts)
Myelodysplastic syndrome, unclassified (MDS–U)	Myelodysplastic syndrome, unclassified (MDS–U)	-

**Table 3:** 2022 WHO and ICC classification systems.

ICC (2022) [27]	WHO–HAEM5 (2022) [27]
Clonal cytopenia of undetermined significance (CCUS)	Clonal cytopenia of undetermined significance (CCUS)
MDS with mutated SF3B1	MDS with low blasts and SF3B1 mutation
MDS with del(5q)	MDS with low blasts and isolated 5q deletion
MDS with mutated TP53	MDS with biallelic TP53 inactivation
MDS, NOS without dysplasia	-
MDS, NOS with single lineage dysplasia	Definition of lineage dysplasia, optional
MDS, NOS with multilineage dysplasia	Definition of lineage dysplasia, optional
-	MDS with low blasts (<5% blasts)
-	MDS hypoplastic
MDS with excess blasts (5–9% blasts)	MDS with increased blasts (MDS–IB1) (5–9% blasts)
MDS/AML (10–19% blasts)	MDS with increased blasts (MDS–IB2) (10–19% blasts)
MDS/AML with mutated *TP53*	-
MDS/AML with myelodysplasia—related mutations	-
MDS/AML with myelodysplasia—related cytogenetic abnormalities	-
MDS/AML NOS	-
-	MDS with fibrosis

Abbreviations: AML, acute myeloid leukemia; MDS, myelodysplastic syndrome/neoplasm; NOS, not otherwise specified, WHO–HAEM4, the World Health Organization (WHO) Classification of lympho-hemopoietic neoplasms, 2016 edition; ICC, International Consensus Classification.

## Data Availability

There were no new data.
